# Cascading effects of habitat loss on ectoparasite-associated bacterial microbiomes

**DOI:** 10.1038/s43705-022-00153-0

**Published:** 2022-08-08

**Authors:** Kelly A. Speer, Tiago Souto Martins Teixeira, Alexis M. Brown, Susan L. Perkins, Katharina Dittmar, Melissa R. Ingala, Claudia Wultsch, Konstantinos Krampis, Carl W. Dick, Spencer C. Galen, Nancy B. Simmons, Elizabeth L. Clare

**Affiliations:** 1 Richard Gilder Graduate School, American Museum of Natural History, New York, NY USA; 2 Center for Conservation Genomics, Smithsonian National Zoological Park and Conservation Biology Institute, Washington, D.C, USA; 3 Department of Invertebrate Zoology, National Museum of Natural History, Smithsonian Institution, Washington, D.C, USA; 4 School of Biological and Chemical Sciences, Queen Mary University of London, London, GBR UK; 5 Department of Ecology and Evolution, Stony Brook University, Stony Brook, NY USA; 6 Sackler Institute for Comparative Genomics, American Museum of Natural History, New York, NY USA; 7 Division of Science, City College of New York, New York, NY USA; 8 Department of Biological Sciences, University at Buffalo, State University of New York, Buffalo, NY USA; 9 Department of Biological Sciences, Fairleigh Dickinson University, Madison, NJ USA; 10 Bioinformatics and Computational Genomics Laboratory, Department of Biological Sciences, Hunter College, City University of New York, New York, NY USA; 11 Institute for Computational Biomedicine, Weill Cornell Medicine, New York, NY USA; 12 Department of Biology, Western Kentucky University, Bowling Green, KY USA; 13 Integrative Research Center, Field Museum of Natural History, Chicago, IL USA; 14 Biology Department, University of Scranton, Scranton, PA USA; 15 Department of Mammalogy, Division of Vertebrate Zoology, American Museum of Natural History, New York, NY USA; 16 Department of Biology, York University, Toronto, ON Canada

**Keywords:** Community ecology, Bacteria, Microbial ecology

## Abstract

Suitable habitat fragment size, isolation, and distance from a source are important variables influencing community composition of plants and animals, but the role of these environmental factors in determining composition and variation of host-associated microbial communities is poorly known. In parasite-associated microbial communities, it is hypothesized that evolution and ecology of an arthropod parasite will influence its microbiome more than broader environmental factors, but this hypothesis has not been extensively tested. To examine the influence of the broader environment on the parasite microbiome, we applied high-throughput sequencing of the V4 region of 16S rRNA to characterize the microbiome of 222 obligate ectoparasitic bat flies (Streblidae and Nycteribiidae) collected from 155 bats (representing six species) from ten habitat fragments in the Atlantic Forest of Brazil. Parasite species identity is the strongest driver of microbiome composition. To a lesser extent, reduction in habitat fragment area, but not isolation, is associated with an increase in connectance and betweenness centrality of bacterial association networks driven by changes in the diversity of the parasite community. Controlling for the parasite community, bacterial network topology covaries with habitat patch area and exhibits parasite-species specific responses to environmental change. Taken together, habitat loss may have cascading consequences for communities of interacting macro- and microorgansims.

## Introduction

Deforestation has well-documented, devastating consequences on species survival [1], global warming [2], and zoonotic disease emergence [3, 4]. This is particularly worrying in tropical forests, which lost 6% of in their global area between 1990 and 2015 [5]. For example, the Atlantic Forest of Brazil, one of the world’s most biodiverse regions, occupies only 28% of its original extent [6]. The consequences of this habitat loss have primarily been examined in macroorganisms, but we do not yet understand the extent to which microorganisms like bacteria, viruses, fungi, and single-cell eukaryotes respond to deforestation, especially those microorganisms that are obligately associated with a living host. These microorganisms are integral members of wildlife communities; changes in their presence or abundance may alter the function of communities [7], the health of individuals and species [8], and the transmission of pathogens between members of the community [9].

**Fig. 1:**
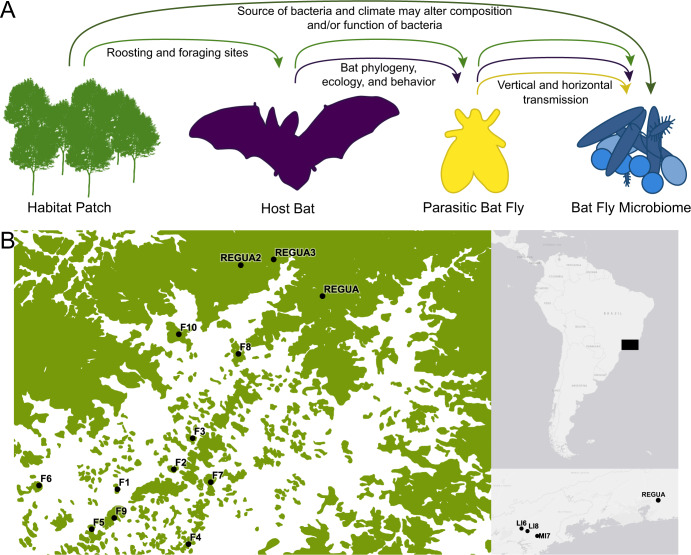
Sampling design. Illustration of how environment (“habitat patch”), host bat, and parasitic bat fly each influence the microbiomes within bat flies (**A**; see in-text description for more details). Sampling map constructed in QGIS v3.12 of REGUA area sites with fragments outside of REGUA labelled with the prefix “F” and ordered from smallest (F1) to largest (F10; **B**), the extent of the sampled area is shaded in black in the map at the top right, and the southern sites are mapped in relation to REGUA in the map on the bottom right. Green area of the map on the left indicates forested habitat based on imagery from SOS Mata Atlántica, while white areas are all non-forested habitat types.

Here, we examine the bacterial microbiome within ectoparasites of bats in forest fragments of varying area and isolation as a model for testing the hypothesis that habitat loss affects host-associated microbial communities (Fig. 1A). We use island biogeography theory as a null hypothesis for the way we expect microbiomes to behave if the environment is driving community composition instead of the host. This theory states that small, isolated habitats will support low-diversity communities that are a subset of the species found in larger source communities [10]. Historically, studies of the island biogeography of parasites have treated hosts as suitable habitat, not the broader environment where the host and parasite live [11, 12]. There are two limitations to this thinking: only an extremely small subset of parasites spend their entire lifespan on the body of a single host individual, and this idea ignores the impact that the environment has on parasite microbiomes that may subsequently impact parasite survival [13]. Hosts are not islands that perfectly constrain their parasites, and parasites are not islands that perfectly constrain their microbes. Previous research suggests that the environment influences microbiome composition following expectations of island biogeography [14, 15]. In other cases, the environment does not dictate microbiome composition [16, 17]. Variation in the ability of the environment to filter members of the microbiome community is a reflection of the complexity and diversity of microorganisms themselves [16, 18]. This diversity makes it difficult to tease apart the impacts of host and environment on microbiome community composition.

Obligate parasites represent a convenient system within which to test potential drivers of microbiome variation for several reasons. The number of factors influencing variation may be more limited in the microbiomes of obligate parasites than in environmental microbiomes (e.g., soil or water) or microbiomes of free-living host species. Unlike free-living host species, obligate ectoparasites have extremely specialized diets, and their movements in the broader environment beyond their host are constrained by their dependence on a host to survive. Ectoparasitic arthropods also have characteristically depauperate microbiome communities compared to arthropods with diverse diets [19]. We can leverage the hierarchical nature of the host-parasite-microbiome system to clearly delimit the microbiome community and restrict the sources of colonizing bacteria that may invade the parasite microbiome, hence providing a manageable system for testing hypotheses about community composition and factors governing assembly of the microbiome.

In this study, we used bat flies (Diptera: Streblidae and Nycteribiidae), which are obligate blood-feeding ectoparasites of bats, to assess community composition of parasite-associated microorganisms across a fragmented landscape in the Atlantic Forest. The microbiome of bat flies may be influenced by the parasitic bat fly, the host bat, and the environment in several ways (Fig. 1A). First, bat fly microbiomes may be vertically inherited or horizontally acquired, as in other insect-microbiome associations (Fig. 1A, yellow line; [20]. Second, bat flies are host-specific and depend on their host for dispersal [21, 22], meaning that the host bat may also influence the bat fly microbiome by altering the community of bat flies from which bacteria may be horizontally acquired (Fig. 1A, dark purple line). For example, bat maternity colonies likely support more abundant parasite communities than bachelor colonies, because bat flies preferentially parasitize female and young bats [23]. By supporting a smaller community of bat flies, bachelor colonies may decrease the sources from which bat flies horizontally acquire bacteria, leading to host-sex-based variation in the microbiome. Other aspects of bat phylogeny, ecology, and behavior may similarly influence bat fly microbiomes, including roost preference, feeding guild, and host bat species identity. Third, habitat patches support a specific bat community based on the availability of roosting and foraging sites, which subsequently alters the diversity and abundance of bat flies [24]. These changes in the local bat fly community may be reflected in associated microbiomes. Lastly, the environment may directly impact bat fly microbiome composition (Fig. 1A, dark green line). Bat flies and all other members of the Hippoboscoidea are adenotrophically viviparous, a condition in which a single egg hatches inside the female fly and the larva feeds from milk glands until it is ready to pupate [21]. In the case of bat flies, the female fly leaves the host bat to deposit the larva on the roost substrate [21], providing opportunities for the environment to act as a source of bacteria for the microbiome of bat flies (Fig. 1A). Deforestation may also increase the local temperature of small habitat patches and directly impact arthropod-associated microbiomes due to thermal constraints of some bacteria [25, 26]. Using the mosaic landscape of the Atlantic Forest, we can examine whether bat fly-associated microbiomes respond to environmental change following island biogeography theory or whether the host bat and parasitic bat fly more strongly determine bat fly microbiome composition.

## Methods

### Sample collection and landscape metrics

Bat flies were collected from bats in 11 habitat patches of the Atlantic forest of Brazil, State of Rio de Janeiro from 18 December 2015 to 19 January 2017 (Table 1, Fig. 1B, Table S1, Fig. S1; [27], including a large protected area of pristine and secondary forest belonging to the Reserva Ecológica de Guapiaçu (REGUA). REGUA is the third largest remaining expanse of Atlantic Forest and was sampled in three separate locations. Samples were additionally collected from three geographically distant habitat fragments (southern sites; Fig. 1B). Each site was sampled for 6 nights, 6 h per night or at least 2 h if there was heavy rain, and between 7 and 10 ground-level mist nets were used to capture bats each night (approximately 60 m of nets were set per night; [27]. Bats were removed from mist nets and placed into freshly washed cloth bags for holding and to minimize cross-contamination of ectoparasites. Each bat was searched for approximately 45 s for ectoparasites, which were captured using featherweight forceps and immediately transferred to tubes containing 92% ethanol, stored at room temperature overnight, and subsequently transferred to −20 °C. Bats were identified in the field following [28, 29]. All capture and handling methods followed recommendations in [30]. Because many bat species were only captured in a subset of sampled sites, we selected bat flies from the six most well-represented bat species for microbiome analysis.

**Table 1:** Sampling of bats and their corresponding flies used for sequencing.

Columns labelled “Total” include female and male flies and flies for which we could not determine a sex due to destruction of the specimen during sampling or transit. We also used flies that had spurious host associations to increase sample size of these species. These are listed under “unknown host.”

Bat flies were identified to species morphologically following [31–33]. Access to comparative morphological material was limited, so we barcoded all samples using *cytochrome oxidase I* [34, 35] (Supporting information; NCBI GenBank accession numbers OL847352−OL847639) and confirmed that individual flies identified morphologically as conspecifics belonged to the same genetic clade.

Environmental variables were measured using ArcGIS 10.1 and Fragstats 3.1 using forest cover maps from the Instituto Brasileiro de Geografia and SOS Mata Atlântica (www.sosmataatlantica.com.br; Table S1; [36, 37]. Habitat fragment area (hectares), isolation (shortest distance between a fragment and its nearest neighbor), distance from source (shortest straight line distance from focal point of a fragment to nearest point of REGUA), perimeter-area ratio, proximity index within a 500 m and 1000 m buffer [36], and forest cover within a 500 m and 1000 m buffer were calculated. REGUA was treated as the source because it is the largest, most biodiverse patch of forest in the study region. Perimeter-area ratio, proximity index, and forest cover were correlated with habitat patch area, isolation, and distance from source, so only these latter three landscape variables were used for downstream analyses. Area, isolation, and distance from source were log_2_ transformed to prevent extremely large or extremely isolated fragments from unduly impacting correlation analyses.

### DNA extraction and 16S rRNA metabarcoding

DNA was extracted from 288 bat flies following a wash step and Proteinase K digestion using the ZymoBIOMICS™ DNA Miniprep Kit (Zymo Research, Irvine, CA, USA) in a Biosafety Cabinet, Class 2 (Supporting information). One negative control was used for each extraction kit to control for laboratory and kit contamination. Negative controls were pooled for amplification. Extracted DNA was aliquoted into 96-well plates for amplification of the hypervariable region 4 (V4) of 16S rRNA following well-documented procedures outlined by the Earth Microbiome Project and the Illumina 16S Metagenomic Sequencing Library Preparation guidelines (Supporting information) [38–41]. Of 288 initial libraries, 77 libraries required an additional concentration step to reach the minimum 2 nM concentration required for sequencing (Supporting information). Low concentration and high concentration libraries were pooled to reach a concentration of 2 nM and sequenced using an Illumina MiSeq v3 Reagent Kit with 2x300bp reads and 18% PhiX spike-in on a MiSeq NGS platform (Illumina, San Diego, CA, USA) at the Bioinformatics and Computational Genomics Laboratory (Hunter College, City University of New York, New York, NY, USA).

### De-mulitplexing, quality filtering, and phylogeny reconstruction

Samples were demultiplexed using the MiSeq Reporter Generate FASTQ workflow. Primer sequences were trimmed from forward and reverse sequence reads using cutadapt v.1.4.2 [42]. Following de-multiplexing, samples were processed using the QIIME2 v.2018.2 pipeline (https://docs.qiime2.org/2018.2) [43–47]. The GreenGenes Database, v.13.5, trimmed to only the 16S rRNA V4 region, was used as a reference to train a naïve Bayes q2-feature-classifier for taxonomic identification of amplicon sequence variants (ASVs) [48]. The GreenGenes Database is not able to discern between “*Candidatus* Aschnera,” the primary symbiont of some nycteribiid flies [49, 50], and *Arsenophonus*, possibly because the V4 fragment of 16S rRNA does not provide enough resolution. To confirm that ASVs of “*Candidatus* Aschnera chinzeii” were not misidentified, we mapped ASVs identified as *Arsenophonus* and “*Candidatus* Phlomobacter” against reference sequences from the Silva Ribosomal Database (Supporting information; [51–53]. Even though the GreenGenes and Silva Databases distinguish between “*Candidatus* Phlomobacter” and *Arsenophonus*, phylogenetic evidence indicates that “*Candidatus* Phlomobacter” is actually a clade nested within *Arsenophonus* [54, 55].

Contamination is ubiquitous in microbiome studies and especially problematic for low biomass samples [56–58]. To reduce the impact of contaminants, several filtering steps were performed (Supporting information). Briefly, we removed bacteria present in negative controls that were likely contaminants (i.e., low relative abundance and low prevalence in samples); known laboratory contaminants [58]; bacteria classified as mitochondria, chloroplast, or Archaea; bacteria unclassified beyond phylum; and contaminants identified by the R package *decontam* v1.14.0 [59–61]. Finally, the data were filtered by two coverage depths: 1) all ASVs present at <0.01% relative abundance within a sample were eliminated from that sample, and 2) all ASVs present at <0.1% relative abundance were eliminated from that sample. At a minimum within-sample relative abundance of 0.01%, spurious ASVs may remain in the dataset, but at a minimum relative abundance of 0.1%, rare ASVs may be incorrectly excluded [62–64].

As low concentration libraries were used to dilute high concentration libraries prior to sequencing, sequencing effort across samples is not even. To assess the bacterial diversity captured by low concentration libraries compared to high concentration libraries, the *ggrare* function was used from the phyloseq-extended suite of tools, which wraps the function *rarefy* from the package vegan v.2.5.4 (https://github.com/mahendra-mariadassou/phyloseq-extended/blob/master/R/graphical_methods.R) [65].

### Data visualization, ordination, and PERMANOVA

We constructed bar plots of the relative abundance of bacterial genera in each bat fly species (*ggplot2*, v.3.1.0) [66, 67]. All genera with a relative abundance <1% of the total reads in a bat fly species were condensed into a “Low Abundance” group. Principal coordinates analysis (PCoA), implemented in *phyloseq* v.1.38.0, was used to visualize differences in microbial communities captured by Euclidean distance between *phylogenetic isometric log-ratio*-transformed relative abundances to account for the compositional nature of metabarcoding data (*philr* v.1.20.0; [66–71], Supporting information).

To test whether landscape variables, parasite variables, or host bat variables were correlated with microbial community composition (i.e., ASV composition and relative abundance), we used PERMANOVA on individual variables and Sequential (Type I) sum of squares on pairs of variables, each with 9999 permutations (*adonis* command in the R package *vegan*) [72, 73]. Homogeneity of dispersion of each group of microbiomes was confirmed using *betadisper* permuted 999 times with *permutest* (R package *vegan*).

When sampling is uneven, sequential sum of squares is sensitive to the order in which variables appear in the equation. To overcome this limitation, we examined the impact of landscape within the four most well-sampled bat fly species, excluding the southern sites and only considering data filtered using a threshold of 0.01% relative abundance per sample. We ordinated samples within these species separately from the rest of the data and estimated variation explained by landscape variables using PERMANOVA on individual variables.

To examine the impact of habitat patch area and isolation on taxon richness, we constructed boxplots of ASV richness by sampling site, mimicking standard island biogeography plots of richness by area, isolation, and distance to the source. We used a Kruskal-Wallis test to assess whether mean richness was significantly different among sampling sites. Spearman correlation was used to examine ASV richness across continuous ranges of ranked area, isolation, and distance from a source.

### Network reconstruction and analysis

Bacterial association networks are a way to visualize correlated changes in relative abundance of bacteria across a given sample set. These networks examine microbiomes at the taxon scale, providing a valuable additional analysis to ordination and PERMANOVA, which compare microbiomes at the community scale. While networks provide a useful tool for further examination of microbiomes, the limitations of networks are still being explored and interpretation of networks should be mindful of known caveats (e.g., bias caused by differing sample sizes). Here, we used networks to explore whether the environment or parasite influence microbiome community structure, taking care to examine the role of sample size in network inference. If the environment drives microbiome composition, then we expect that networks will exhibit conserved changes in structure as habitat patches decrease in area or increase in isolation. If parasite species identity impacts microbiome composition, we expect that network structure will be specific to parasite species but will not vary with habitat patch area or isolation.

Networks were reconstructed using *SPIEC-EASI* v.1.1.2 using the Meinshausen and Bühlmann method [74]. Nodes indicate individual bacterial ASVs and edges (i.e., connections between nodes) indicate a linear relationship in the abundances of linked nodes. *SPIEC-EASI* transforms the relative abundance of each ASV using centered log-ratios and then estimates an inverse covariance matrix by solving a regularized linear regression for each node to determine its conditional independence within the graph [74]. Two nodes are conditionally independent when their abundances are statistically independent given the abundances of all other nodes in the network. The λ tuning parameter is used to penalize linear regressions and controls the sparsity of the final network. To select the sparsity of the final network, *SPIEC-EASI* builds graphs from repeated subsamples of the data (i.e., Stability Approach to Regularization Selection [75]), and selects the λ value that yields the greatest stability of edge incidences across subsampled graphs. The *SPIEC-EASI* method is distinct from network estimations based on correlation or covariance, which rely on pairwise comparisons and may incorrectly infer an edge between indirectly linked nodes by ignoring the influence of other nodes in the network.

Two types of networks were reconstructed: habitat patch networks estimated from all samples within a single sampling site (1 network per site), and species-specific networks estimated from well-represented parasite species clustered by their occurrence within or outside of REGUA (1 network of within-REGUA samples and 1 network of outside-REGUA samples for each parasite species; Supplemental information). The *lambda.min.ratio* parameter was adjusted until network stability was within 0.002 (habitat patch networks) or 0.003 (species-specific networks) of the target 0.05 threshold, then *nlambda* was set to either 20, 30, or 50 [74, 75]. To examine the impact of sample size on habitat patch networks, we leveraged the subsampling scheme within SpiecEasi to test whether the proportion of highly confident edges (i.e., edges present in at least 80% of sampled networks) was correlated with sample size using the *getOptMerge* function.

Characteristics of networks provide information about the robustness of a community and the function of members of a community [76]. Leading eigenvector modularity of each network and betweenness centrality of each node were estimated in the R package *igraph*, v.1.2.11 [77–80]. Modularity is a measure of the structure of a network, where higher modularity indicates nodes are grouped into tightly interacting neighborhoods with few interactions occurring outside of this neighborhood [81]. Leading eigenvector modularity is an optimization method of community detection with known limitations, despite being widely used [82]. It is unclear how this method performs on sparse, disconnected networks, but modularity estimates may be noisy or difficult to resolve [83]. Betweenness centrality measures the number of times the shortest path between all pairs of nodes in the network travel through a given node, giving an estimate of the influence of a node on the structure of the network [81]. Network connectance is the proportion of realized edges relative to the total possible number of edges [81]. We examined the correlation of modularity, betweenness centrality, and connectance with landscape variables and used Mann-Whitney U and Kruskal-Wallis tests to test for significant differentiation.

Modularity and betweenness centrality are impacted by network size (i.e., number of edges) and shape (degree distribution; [81]), making comparisons of summary statistics between networks inaccurate. To account for variation in network size and shape in habitat patch networks, we used several standardization techniques to compare networks: 1) centered modularity compared to mean modularity of patch-specific null distribution; 2) Z-score modularity compared to a patch-specific null distribution; 3) Z-score modularity compared to the mean modularity of measured networks (see Supporting information for details on null distribution).

As a size-independent method of examining variation between networks, we used the graphlet correlation distance from [84, 85] to ordinate the networks as individual points on a plot using the R packages *pulsar* v.0.3.5 and *orca* v.1.1-1 [86, 87]. The graphlet correlation distance breaks up a network into up-to 4-node graphlets (i.e., all the possible ways that up to 4 nodes can be wired) and counts the number of times each node plays a specific role within a graphlet (i.e., orbit). For example, within a 3-node graphlet where two “leaf” nodes interact with a central node but not each other (i.e., a line of 3 nodes), the “leaf” nodes belong to one orbit and the central node belongs to the second orbit in this graphlet. Using these orbit counts, we estimate the Spearman correlation of orbits across all nodes. Following [88, 89], the Euclidean distance between these correlations can be used to ordinate the networks and more clearly visualize their differentiation. We used k-means clustering to distinguish groups of networks in ordination space.

## Results

Of 228 prepared DNA libraries 222 libraries were used for downstream analysis following quality filtering (NCBI SRX13352735−13352962; BioProject PRJNA786937). Filtered libraries ranged in sequencing depth from 2983 to 66,164 reads. A total of 1155 ASVs were detected when a 0.01% filtering threshold was applied, while 526 ASVs were found under a 0.1% filtering threshold. Rarefaction curves showed a plateaued asymptote for each library and low concentration libraries fell within the range of ASVs detected in high concentration libraries generated from the same parasite species (Figs. S2 and S3).

### Composition of sampled bat fly microbiomes

**Fig. 2:**
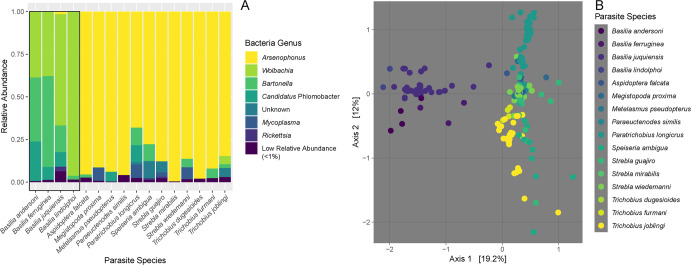
Bat fly microbiome composition. Relative abundance of each bacterial genus summed across repeated samples of each parasite species (**A**). Colors indicate different bacterial genera and bars represent each parasite species. The black box surrounds the nycteribiid bat flies. Low abundance bacteria were those comprising less than 1% relative abundance in each species. Unknown bacteria could not be identified to genus using the GreenGenes database. Principal Coordinates Analysis on the Euclidean distances between philr-transformed microbial abundances of the complete dataset (**B**). Colors represent parasite species.

Plots of relative abundance of bacterial genera showed a stark difference between the microbiome communities in the parasite families Nycteribiidae and Streblidae (Fig. 2A; Fig. S4). While nycteribiid bat flies had high relative abundances of *Wolbachia* and *Bartonella*, streblid bat flies were dominated by *Arsenophonus*. We did not detect “*Candidatus* Aschnera chinzeii” and *Arsenophonus* (including “*Candidatus* Phlomobacter”) was detected in low relative abundance in nycteribiid bat flies. Almost no *Wolbachia* was detected in streblid bat flies. *Bartonella* was present in some streblid bat flies, but at much lower relative abundance than in nycteribiid bat flies. *Mycoplasma* was also detected at higher relative abundances in streblid bat flies compared to nycteribiid flies. The flies in southern fragments were dominated by *Wolbachia* and *Bartonella*, likely due to the abundance of nycteribiid flies in these fragments (Fig. S5).

### Variation in the microbiome in response to parasite, bat host, and environment

Ordination of microbiomes and PERMANOVA indicated that the parasite (i.e., parasite family and species), the host bat (i.e., bat family, bat sex, and bat individual), and the environment (i.e., region and sampling site) significantly contributed to bat fly microbiome variation (Table 2; Fig. 2B and Fig. S6). Other variables significantly contributed to microbiome community differentiation (i.e., bat feeding guild, bat species, protection status of sampling site, habitat patch area, and isolation), but violated PERMANOVA’s assumption of homoscedasticity. Parasite species, parasite family, bat feeding guild, bat family, bat species, and sampling site had the largest effect sizes, however many of these variables are correlated with each other.

**Table 2:** Univariate PERMANOVA analysis of parasite, bat, and landscape influences on microbiome variation.

Results are provided for the full dataset (all sites at 0.01% threshold for bacterial relative abundance per sample), the strictly filtered dataset (all sites at 0.1% threshold for bacterial relative abundance), the samples collected from REGUA area sites (excluding southern sites), and the samples collected from fragments outside of REGUA (excluding southern sites). Grey shading indicates variables that significantly differentiated microbiomes and did not violate the assumptions of homoscedasticity.

Univariate PERMANOVA results indicating the *p* value (top; * = *p* < 0.05, ** = *p* < 0.005, *** = *p* < 0.0005), R2 (middle) and *p* value for homoscedasticity (bottom, significance indicates violation of the assumptions of PERMANOVA).

Sequential sum-of-squares with free permutation indicated that parasite species significantly impacted microbiome community structure, consistent with single variable PERMANOVA (Table 3). Habitat patch protection status and region also significantly contributed to microbiome variation. In the four most well-sampled parasite species, none of the test variables significantly explained microbiome variation without violating PERMANOVA assumptions (Table S2; Fig. S7).

**Table 3:** Sequential Sum-of-Squares where the impact of each variable is considered after the impact of parasite species is accounted.

Grey shading indicates significant impact without violating assumptions of PERMANOVA. The PERMANOVA *p* value (top; * = *p* < 0.05, ** = *p* < 0.005, *** = *p* < 0.0005), R2 (middle) and *p* value for homoscedasticity (bottom, significance indicates violation of the assumptions of PERMANOVA) are provided for each dataset.

### Bacterial taxon richness across habitat patches

**Fig. 3:**
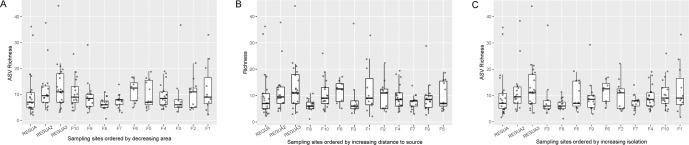
Taxon richness by site. Box and whisker plot of the bacterial ASV richness in each sampling site in order of decreasing area (**A**), increasing distance from a source (**B**), and increasing isolation (**C**).

While the three sampled sites within REGUA had the highest ASV richness, there was no pattern of decreasing ASV richness with decreasing area, increasing isolation, or increasing distance from a source (Spearman; Area: rho = 0.0613, *p* value = 0.4031; Isolation: rho = 0.0020, *p* value = 0.9787; Distance to Source: rho = −0.0564, *p* value = 0.4421; Fig. 3). Median bacterial ASV richness fell between 6 and 11 for each sampled parasite individual, but the range of ASV richness per fragment varied dramatically. There was no significant difference between sampling sites in mean ASV richness (Kruskal-Wallis chi-squared statistic = 17.856, *p* value = 0.1201).

### Bacterial association networks

**Fig. 4:**
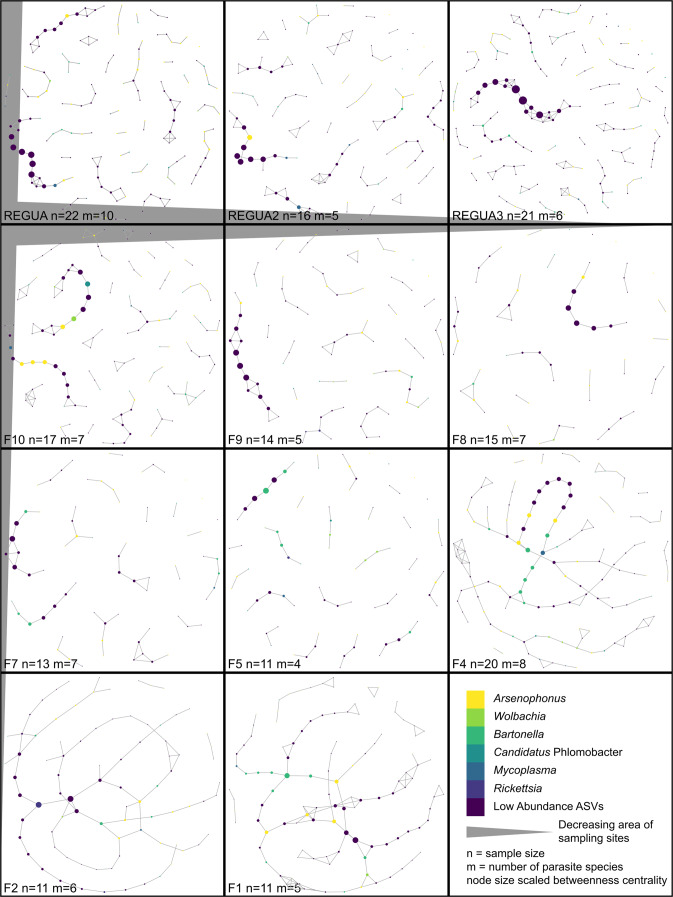
Bacterial association networks. Networks for each sampling site are ordered by decreasing habitat patch area, with the largest sites in the top left and the smallest sites in the bottom right. The size of the nodes in the networks corresponds to the z-score betweenness centrality of that node scaled by the range of betweenness centralities detected within the network.

**Fig. 5:**
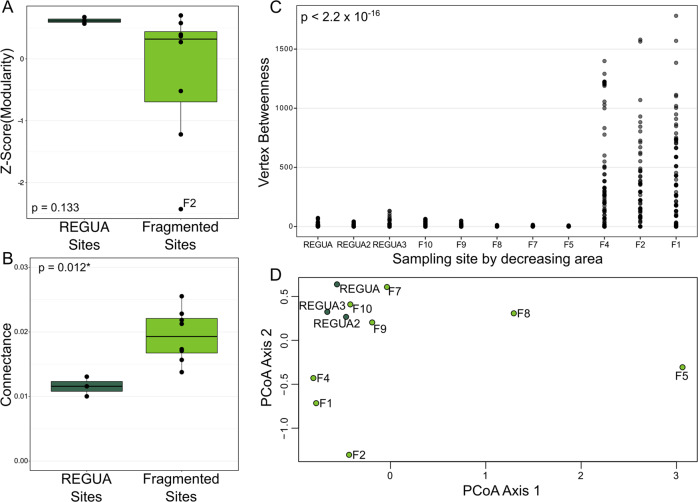
Summary statistics of network plots. Z-score modularity of habitat patch networks between REGUA and outside-REGUA sites (**A**), network connectance of habitat patch networks in REGUA and outside-REGUA sites (**B**), vertex betweenness centrality by fragment decreasing by habitat patch area (**C**), principal coordinates analysis of the distribution of network orbits for habitat patch networks (**D**). Dark green corresponds to REGUA sites and lime green corresponds to sites outside of REGUA for which networks were reconstructed.

The connectance and betweenness centrality of habitat patch networks varied with habitat patch effects, but modularity did not (Figs. 4 and 5). Network connectance was significantly lower in REGUA patches than in networks constructed for sites outside of REGUA (Fig. 5B; Wilcoxon Rank Sum test, *p* value = 0.012). Betweenness centrality was higher in networks for patch F4, F2, and F1 (i.e., smallest patches), corresponding with their band-like network structure (Fig. 4), and centrality was significantly different between networks from large and small patches (Fig. 5C; Kruskal-Wallis chi-squared statistic = 300.23, *p* value <2.2 × 10^−16^). Raw and Z-score modularity of habitat patch networks did not significantly differ between REGUA and non-REGUA sites, but tended to be lower in patches outside REGUA (Fig. S8). Z-score modularity using the measured networks for standardization did not control for network size and shape, and mimicked the pattern exhibited by raw modularity. Modularity measures standardized by null distributions did not support patterns of decreasing modularity with decreasing patch area (Fig. S8).

In habitat patch networks reconstructed for the three REGUA sites and large patches, low-abundance bacteria had higher betweenness centrality than other bacteria in these networks, acting to connect graph neighborhoods (Fig. 4). As habitat patch networks became less modular with decreasing habitat patch area, nodes with high betweenness centrality were typically low-abundance bacteria and bacteria in the genera *Arsenophonus* (including *Candidatus* Phlomobacter), *Wolbachia*, and *Bartonella*.

While there was no impact of sample size (i.e., number of parasite individuals) on ASV richness in each network, a greater number of samples may allow detection of more edges between nodes and change the size of a network. Sample size was lowest in small fragments and highest in large fragments, with the exception of F4 which has intermediate area, isolation, and distance to source measurements, but supports a high diversity and abundance of parasites. We did not detect a significant correlation between sample size and network modularity (Spearman rho = 0.3945, *p* value = 0.229). Using the subsampling scheme within SpiecEasi, we did not detect a correlation of sample size and proportion of high-confidence network edges (Spearman rho = 0.0275, *p* value = 0.936).

Using ordinations of graphlet orbits as a size-independent comparison of networks [84, 88, 89], habitat patch networks vary with habitat area (Fig. 5D) and species-specific networks are distinct within and outside of REGUA (Fig. S8). Principal coordinate axis 2 corresponded well to decreasing habitat area, with large fragments positioned higher on the access and gradually decreasing in patch area lower on the axis. Principal coordinate axis 1 primarily illustrated variation in networks from patches F8 and F5. This variation does not correspond with environment, parasite, or bat variables. K-means clustering with 3 groups separated large habitat patch networks (REGUA networks, F10, F9, F7) from small patch networks (F4, F2, F1), and F8 and F5 formed a unique cluster. Species-specific networks also indicated distinctions between within-REGUA and outside-REGUA networks. All within-REGUA networks occupied unique ordination space from outside-REGUA networks within species.

## Discussion

Our research builds upon previous evidence that the environment influences microbiome composition in addition to host factors [90–93]. However, previous studies have been primarily conducted on the microbiomes of free-living hosts or microbiomes within a single host species, preventing the examination of how the environment might impact communities of interacting macro- and microorganisms. Contrary to expectations, habitat loss did not lead to a decrease in bacterial diversity. Instead, habitat loss impacted bacterial community structure, which includes diversity and relative abundance. The interactions of bat flies with the broader environment are filtered through their obligate associations with host bats, yet the signal of environmental change is also detected in the composition of bat fly microbiomes. This indicates that environmental degradation may have cascading consequences through hierarchical communities of interacting organisms.

### Parasite and environment as drivers of microbiome composition

Parasite species identity is the strongest predictor of microbiome composition (Tables 2 and 3; Fig. 2B). Specificity of arthropod microbiomes has been previously found in tsetse flies (Glossinidae; [94], which are also members of the Hippoboscoidea. That microbiome composition shows such a strong signal of parasite species identity indicates that either more of the microbiome is maternally inherited than previously understood, or that even non-maternally inherited bacteria may be maintained through life history traits (e.g., host bat associations, microclimatic preference; horizontal transmission of bacteria).

Habitat patch area and protection status, but not degree of isolation or distance to source, have a measurable effect on the microbiome of bat flies, but less explanatory power than parasite species (Tables 2 and 3; Figs. 3–5). While bacterial ASV richness does not vary following expectations of island biogeography theory (Fig. 3), examining both relative abundance and diversity of bacteria using PERMANOVA and bacterial association networks provided a clear statistical signal that habitat patch area (measured as area and protection status) is correlated with microbiome composition (Table 3; Fig. 5). Other measures like isolation and distance to source had no impact on bat, parasite, or microbiome communities, likely because bats can easily move between these patches. However, even when habitat patches are proximal, fragmented landscapes in the Atlantic Forest have been previously shown to significantly impact bat–plant and bat–ectoparasite interaction networks [95].

Even though changes in the bat and parasite communities explain most of the variation in microbiome association networks, the environment may also directly impact bat fly microbiome communities. The structure of habitat patch networks covaried with habitat area and parasite species-specific networks were consistently different within REGUA compared to outside REGUA. In addition, parasite and bat species diversity did not always correspond to a specific network structure. REGUA and fragments F10 (the largest fragment outside of REGUA, 9 bat fly species) and F4 (8 bat fly species) have the greatest parasite richness, but F4 is intermediate in area and isolation. Networks from patch F4 consistently cluster with other networks from small patches (Fig. 5C, D), despite having similar parasite species richness to large fragments. That patch F4 has high parasite species richness but similar bacterial association networks to smaller fragments (i.e., lower parasite species richness), suggests that bat and parasite community composition does not solely explain microbiome variation. The environment may also directly drive composition of parasite-associated microbiomes.

### Implications of changes in bacterial network structure in response to environment

Bacteria with high betweenness centrality may act as hub species that maintain the stability of a network [19, 92, 93]. In small fragments, *Arsenophonus*, *Wolbachia*, and *Bartonella* had high betweenness centrality, but these bacterial taxa were less central to the networks from large fragments despite maintaining high relative abundance in flies at these sites. Decreasing betweenness centrality may be indicative of changing interactions between bacteria in response to environmental perturbations. Bacteria of blood-feeding insects play an important role in vector competence in insects [19, 96, 97]. For example, in tsetse flies, primary bacterial endosymbionts in the genus *Wigglesworthia* impede the invasion of trypanosome parasites by assisting host defenses and subsequently decrease the competence of tsetse flies to vector these harmful parasites to downstream hosts including humans [98]. As bat flies are important arthropod vectors of bat pathogens [99], changes in the structure of their microbiomes in response to habitat loss may have implications for the disease ecology of arthropod vectored pathogens in bats.

Modules may delimit groups of bacteria with specific functional specializations and/or groups that respond in similar ways to environmental variables [99, 100]. Higher modularity may protect a community of free-living organisms from invading pathogens, because a pathogen would be isolated to one module within the community (i.e., diversity-stability debate) [100, 101]. This hypothesis may be applicable to bacterial networks if pathogens are limited in transmission by direct competition with endogenous bacteria. However, high modularity in bacterial association networks may also reflect the absence of microbiome-mediated host defenses against pathogen invasion. If we revisit the tsetse fly example, *Wigglesworthia* and trypanosome parasites would share an edge in a microbe association network because *Wigglesworthia* abundance and prevalence is associated with low trypanosome abundance and prevalence mediated by host defenses. The absence of this interaction in a network might indicate that *Wigglesworthia*-induced host response to trypanosome invasion is impaired by other microbes in the network (e.g., priority effects) or other aspects of host health. Trypanosome transmission would be less regulated in this instance, but modularity would be high as long as trypanosome abundance and prevalence did not lead to changes in the abundance or prevalence of other microbes. In the case of bat flies, the consequences of more isolated modules for the functionality and stability of the microbiome are unclear and merit future investigation.

### The missing primary symbionts of Brazilian nycteribiid flies

*Arsenophonus* has been previously identified as a primary symbiont of streblid bat flies and the closely-related “*Candidatus* Aschnera chinzeii” is the hypothesized primary symbiont of some nycteribiid bat flies [49, 50, 102–104]. The high relative abundance and prevalence of *Arsenophonus* in all streblid bat flies sampled in our study is consistent with the hypothesis that *Arsenophonus* acts as the primary symbiont in the family Streblidae. However, it is unlikely that *Arsenophonus* or “*Candidatus* Aschnera chinzeii” are acting as the primary symbionts of the nycteribiid species that we sampled (Fig. 2A). Previous studies that have identified “*Candidatus* Aschnera chinzeii” as the primary symbiont in nycteribiid flies examined species that are geographically limited to Africa, Asia, Europe, and Oceania [102, 103]. Species within *Basilia*, the only globally distributed bat fly genus [105, 106], have varying symbiont associations [49, 50, 103, 104]. Only one previous study has examined symbionts of *Basilia* from The Americas [107]. This study detected an *Arsenophonus* variant in two individuals of *Basilia boardmani* (Nycteribiinae; restricted to North America) that was distinct from “*Candidatus* Aschnera chinzeii” and from *Arsenophonus* detected in other nyteribiid species. In the *Basilia* species sampled from The Atlantic Forest, of which 3 are limited to South America (*B. andersoni*, *B. juquiensis*, *B. lindolphoi*) and 1 is found in North and South America (*B. ferruginea*), we detect no “*Candidatus* Aschnera chinzeii” and low relative abundance or an absence of *Arsenophonus* (including “*Candidatus* Phlomobacter”). These findings in combination with previous studies suggest that neither “*Candidatus* Aschnera chinzeii” nor *Arsenophonus* act as the primary symbiont of the sampled *Basilia* species. It may be that *Wolbachia* and *Bartonella* both have high relative abundance in nycteribiid bat flies because they are acting as primary or facultative symbionts, or because they are acting as a reproductive parasite and pathogen, respectively [107–113]. If the latter is true, the primary symbiont may be one of the less abundant bacteria or another microbe not detected by 16 S rRNA sequencing (e.g. fungi; [114].

### Summation

Understanding of community structure and function must be extended beyond an exclusively macroorganismal view to include the layers of microorganisms that also participate in defining an ecological community. By examining the hierarchical interactions between bats, bat flies, and bat fly bacterial microbiomes within a largely deforested landscape, we attempted to more accurately characterize the consequences of environmental change for a wildlife community. Reduced fragment area led to generally less diverse and less abundant bat fly communities, which led to less modular bacterial association networks, but without a decrease in bacterial ASV richness. Our data highlight the importance of considering ecological responses of microorganismal taxa. Future research is needed in order to capture the full impact of continuing deforestation and habitat change on ecological communities.

## Supplementary information

Supplementary Information

## Data Availability

Raw 16S rRNA reads are available under NCBI SRA accessions SRX13352735−13352962 and BioProject PRJNA786937, COI sequences of bat flies are available on GenBank (OL847352−OL847639), metadata and all code used for analyses is available on Github (10.5281/zenodo.6796123).
