# Supplementary material for: Cascading effects of habitat loss on ectoparasite-associated bacterial microbiomes
Source: ISME Commun. 2022 Aug 8;2:67. doi: 10.1038/s43705-022-00153-0 (PMC9723575; doi:10.1038/s43705-022-00153-0)
Supplement: Supplementary file 1 — Supplementary Information [file 43705_2022_153_MOESM1_ESM.pdf]

Supplementary Information for

Cascading effects of habitat patch area on ectoparasite communities and their associated bacterial microbiomes

SUPPLEMENTAL METHODS

DNA extraction

Flies were separated into individual tubes and washed twice by suspending in 500µL phosphate-buffered-saline (1x) and vortexing to dilute exoskeletal bacterial contamination. Following washing, the abdomen of each fly was separated from the thorax using sterile forceps and proteinase K was used to digest soft tissue from the entire fly overnight following manufacturer instructions (55°C). Extractions followed manufacturer protocol with the following exceptions: samples were bead beat in a Disruptor Genie for 20 minutes at 3000 rpm (max speed); following bead beating, samples were stored at -80°C following manufacturer guidelines; sterile water used for elution of DNA from the filter was heated to 55°C; the elution incubation step was increased to 5 minutes; and the elution step was repeated using the first eluate to re-hydrate the column filter.

16S rRNA Library Preparation: Amplification and Indexing Reactions

Earth Microbiome Project primers were employed with Illumina overhangs for barcoding Primers 515f (TCGTCGGCAGCGTCAGATGTGTATAAGAGACAGGTGYCAGCMGCCGCGGTAA) and 806r (GTCTCGTGGGCTCGGAGATGTGTATAAGAGACAGGGACTACNVGGGTWTCTAAT) with Illumina overhang were used for amplification of the V4 region of 16S rRNA (Gilbert et al. 2010; Gilbert, Jansson, and Knight 2014; Apprill et al. 2015; Parada, Needham, and Fuhrman 2016). Amplicon PCRs were performed in triplicate 25µL final reaction volumes containing 10µL 5PRIME HotMasterMix (final concentrations of 1U Taq DNA Polymerase, 45mM Cl, 2.5mM Mg_2+, 200µM of each dNTP; Quantabio, Beverly, MA,USA), 5µL of each primer at 1µM concentration (final concentration of 0.2µM each), and 5µL of template DNA. Thermocycler conditions used an initial denaturation of 94°C for 2 minutes, followed by 30 cycles of 94°C for 20 seconds, 55°C for 30 seconds, and 65°C for 30 seconds, with a final elongation step at 65°C for 5 minutes and a storage temp of 4°C. Extraction negative controls were pooled into a single aliquot and an additional negative control was introduced during PCR amplification. Triplicate PCRs were combined and then cleaned using SPRIselect magnetic beads following manufacturer's instructions (Beckman Coulter, Sykesville, MD, USA). Concentration of cleaned PCR products was estimated using the Qubit 2.0 fluorometer dsDNA HS Assay Kit (Invitrogen, Carlsbad, CA, USA), and 10% of samples were run on a Bioanalyzer 2100 DNA High Sensitivity chip (Agilent, Santa Clara, CA, USA) to assess representative quality of preparations and verify consistency in amplicon sizes. Indexing PCRs were conducted in 50µL final reaction volumes containing 25µL KAPA HiFi HotStart Ready Mix (final concentrations of 0.5U Taq DNA polymerase, 2.5mM $MgCl_2$, and 0.3mM of each dNTP; KAPA Biosystems, Wilmington, MA, USA), 5µL of each the forward and reverse indexing primers (Illumina Nextera XT Index Kit v2, set A, set B, and set C), and 5µL clean amplicon PCR product, following recommendations in the Illumina 16S Metagenomic Sequencing Library

Preparation guidelines. Thermocycler conditions used an initial denaturation at 95C for 3 minutes, followed by 8 cycles of 95C for 30 seconds, 55C for 30 seconds, and 72C for 30 seconds, finishing with a final elongation at 72C for 5 minutes and a 4C holding temperature. Indexed libraries were cleaned using SPRIselect magnetic beads and concentration and quality of libraries was estimated as described above.

16S rRNA Library Concentration and Pooling

Libraries that were lower than 2nM were concentrated using SPRIselect magnetic beads to remove 10mM Tris pH 8.5 and vacufuged until dry. Libraries were re-hydrated with 4-6µL sterile water depending on initial concentration, and concentration and quality were re-assessed using Qubit and Bioanalyzer. Following concentration of low-yield samples, 206 equimolar libraries were combined into a 3.4nM "high concentration pool" and 23 libraries at a concentration of less than 3.4nM were combined into a single 1nM "low concentration pool", which was used to dilute the "high concentration pool" from 3.4nM to 2nM for sequencing.

Barcoding of bat flies

To barcode the bat flies for which microbiomes were sequenced, a 710bp fragment of COI was amplified from extracted DNA using universal primers developed by (Folmer et al. 1994); LCO1490: 5'-GGTCAACAAATCATAAAGATATTGG-3', HCO2198: 5'-TAAACTTCAGGGTGACCAAAAAATCA-3'). PCRs were conducted in 15µL reactions using 7.5µL 2x TopTaq Master Mix (Qiagen, Hilden, Germany); 0.1µM final concentration of each the forward and reverse primer, 1.5µL of 10x Coral Load (Qiagen, Hilden, Germany), and 1µL of template DNA, with the remaining volume filled by sdH_2O. Thermocycler conditions followed (Hebert et al. 2003). The success of PCRs was confirmed using gel electrophoresis (1.5% agarose gel). PCRs were cleaned using AMPure XP beads (Beckman Coulter, Indianapolis, IN) following manufacturer instructions. Cycle sequencing reactions were conducted in 10µL reactions containing 1µL Big Dye Terminator v3.1 (Life Technologies Corporation, Carlsbad, CA, USA), 1µL extension buffer, 0.1µM final concentration of primer, 2µL of cleaned PCR product, and sdH_20 to the final volume. Thermocycler conditions were as follows: initial denaturation at 94C for 5min, 25 cycles of denaturation at 94C for 40s, annealing at 50C for 30s, and elongation at 60C for 4min, and holding at 10C. Cycle sequencing reactions were cleaned using ethanol precipitation and rehydrated with DNA Injection Solution (Montage, Temecula, CA, USA) for sequencing on the ABI 3730xl DNA Analyzer at the Sackler Institute of Comparative Genomics at the American Museum of Natural History. Sequence chromatographs were trimmed to a 645bp segment, checked for quality, and aligned using ClustalW in Geneious v.10.2.4 (Thompson, Higgins, and Gibson 1994; Larkin et al. 2007; Kearse et al. 2012). A phylogeny was reconstructed using RAxML v.8 assuming a model of evolution of GTR+G, based on AIC scores from jModelTest 2.1, with 1000 bootstrap replicates on the CIPRES Science Gateway (Miller, Pfeiffer, and Schwartz 2010; Darriba et al. 2012; Stamatakis 2014). The phylogeny was examined in FigTree v.1.4.2 to confirm clades established from morphological identifications of bat flies (Rambaut and Drummond 2012; Rambaut 2014).

QIIME2 processing

Following de-multiplexing, samples were processed using the QIIME2 v.2018.2 pipeline (https://docs.qiime2.org/2018.2/). DADA2 was used to filter out PhiX reads and chimeras, truncate the length of reads (forward = 200bp, reverse = 180bp), and cluster reads into unique

amplicon sequence variants (ASVs) corrected for Illumina sequencing errors (Callahan et al. 2016). Reads were aligned using the MAFFT plugin in QIIME2 (FFT-NS-i;(Katoh et al. 2002, 2005; Katoh and Toh 2007). Default parameters were used to mask highly variable regions of the alignment and reconstruct a phylogeny using the FastTree2 plugin (Price, Dehal, and Arkin 2010), which was midpoint-rooted. The GreenGenes Database, v.13.5, trimmed to only the 16S rRNA V4 region, was used as a reference to train a naïve Bayes q2-feature-classifier for taxonomic identification of ASVs (DeSantis et al. 2006).

Filtering 16S rRNA data for contamination

First, any bacterial taxon detected in the negative controls was removed from all other samples, with the exception of *Arsenophonus*. This genus of bacteria contains known symbionts of insects and is expected to be associated with bat flies (Trowbridge, Dittmar, and Whiting 2006; Nováková, Hypsa, and Moran 2009). As *Arsenophonus* is highly abundant in the samples sequenced for this study, it may be that its detection in the extraction control (0.7% of 3,524 total reads) and PCR control (55% of 63 total reads) is due to index bleed, a known issue when multiplexing samples (Eisenhofer et al. 2019), and it is not treated as a contaminant here. Next, bacterial genera were removed that are known laboratory contaminants (Eisenhofer et al. 2019), as were reads that were classified as being derived from mitochondria, chloroplast, or Archaea, or those that could not be classified beyond phylum. Data were exported from QIIME2 and reformatted for import into the R package *phyloseq* v.1.26.1 (McMurdie and Holmes 2012, 2014) for further decontamination and all downstream analyses. We used the R package *decontam* to identify ASVs whose frequency is inversely correlated with initial library concentration (Davis et al. 2018). Nine additional ASVs were identified as potential contaminants and eliminated from the dataset. *Arsenophonus* was not identified as a contaminant by *decontam*.

Reference Database for "Candidatus Aschnera chinzeii"

The GreenGenes Database does not include "*Candidatus* Aschnera chinzeii", a close relative of *Arsenophonus* that has previously been identified as the primary symbiont of some nycteribiid flies (Hosokawa et al. 2012; Duron et al. 2014). To identify reads belonging to "*Candidatus* Aschnera chinzeii," we built a custom BLAST database containing reference sequences for "*Candidatus* Aschnera chinzeii" (N=4), *Arsenophonus* (N=37), and "*Candidatus* Phlomobacter" (N=3; Silva Ribosomal RNA Database; (Quast et al. 2013; Yilmaz et al. 2014; Glöckner et al. 2017) against which we compared all 16SrRNA ASVs that were classified as *Arsenophonus* by the naïve Bayes classifier.

Transformation, ordination, PERMANOVA of compositional data

Metabarcoding using high-throughput sequencing is compositional in nature – meaning the total observations (reads per ASV) for a sample contain no information about the total number of microbes and is dependent on the sequencing capacity of the instrument (Fernandes et al. 2014; Gloor and Reid 2016; Gloor et al. 2017; Tsilimigras and Fodor 2016; Xia and Sun 2017). To correct for the compositional nature of 16S rRNA sequencing data, isometric log-ratio transformations were implemented in the R package *philr* v.1.8.1 (Silverman et al. 2017). This transformation utilizes a user-provided bacterial phylogeny to standardize the abundance of bacterial taxa in a sample by the abundance of its sister taxon, creating "balances" at each node on the phylogeny (Silverman et al. 2017). Euclidean distances between *philr* balances provide

phylogenetic and abundance information about the bacteria in a sample, similar to weighted UniFrac, that can be used for ordination and down-stream statistical analysis (Gloor et al. 2017; Silverman et al. 2017).

As this is a nested system (within each fragment, we expect to see a subset of bat species, and within each bat species, only a subset of bat flies occur, and within each bat fly only a subset of bacterial taxa occur), assessing each variable separately ignores the interactions that could impact our conclusions. Sequential (Type I) sum of squares was used to account for the nonindependence of variables in testing for significant differentiation between microbiome communities. In each test, parasite species was the first variable, followed by one additional variable, and the interaction between parasite species and the additional variable (e.g., pairwise sample distance matrix ~ parasite species + log-scaled area + the interaction between parasite species and log-scaled area). The additionally examined variables were bat species, bat sex, bat individual, region (REGUA area or southern sites), log2-scaled area, log2-scaled isolation, distance from source, protection status (within REGUA or outside of REGUA, excluding the southern sites), and sampling site. PERMANOVA analyses were performed on a dataset containing all localities with taxa filtered at 0.01% relative abundance per sample, a dataset containing all localities with taxa filtered more strictly at 0.1% relative abundance per sample, only the REGUA area localities (no southern sites), and only unprotected REGUA area localities (no localities within REGUA).

Bacterial interaction network reconstruction and analysis

Southern fragments were excluded from all network analyses and REGUA area fragments F3 and F6 were excluded from the habitat fragment networks, because they had fewer than 10 samples. Species-specific networks were reconstructed for well-sampled parasite species. A within-REGUA and an outside-of-REGUA network was reconstructed for each *Aspidoptera falcata*, *Basilia juquiensis*, *Paratrichobius longicrus*, and *Strebla wiedemanni*. Only an outside-of-REGUA network was constructed for each *Strebla guajiro* and *Trichobius joblingi* because sample sizes were too low to estimate networks for these species within REGUA. The within-REGUA samples of *Strebla wiedemanni* were filtered so that only taxa that occurred at least 10 times (summed across all samples used in the network) were maintained, so that the network would reach stability.

To control for network size and shape, we created a null distribution for each habitat fragment network of 100 randomly re-wired graphs with degree distribution preserved. For each random network, the number of rewiring trials performed was equal to ten times the total number of nodes in the network. We centered the modularity of each measured network by the mean modularity of its corresponding null distribution. We also calculated the Z-score modularity using the mean and standard deviation of the measured networks (e.g., [modularity of F1 network − mean modularity of all networks]/standard deviation of modularity of all networks) and the Z-score modularity using the mean and standard deviation of each null network (e.g., [modularity of F1 network − mean modularity of F1-specific null distribution]/standard deviation of F1-specific null distribution).

Literature Cited

Apprill, A., S. McNally, R. Parsons, and L. Weber. 2015. "Minor Revision to V4 Region SSU RRNA 806R Gene Primer Greatly Increases Detection of SAR11 Bacterioplankton." *Aquatic Microbial Ecology: International Journal* 75 (2): 129–37.

Callahan, Benjamin J., Paul J. McMurdie, Michael J. Rosen, Andrew W. Han, Amy Jo A. Johnson, and Susan P. Holmes. 2016. "DADA2: High-Resolution Sample Inference from Illumina Amplicon Data." *Nature Methods* 13 (7): 581–83.

Darriba, Diego, Guillermo L. Taboada, Ramón Doallo, and David Posada. 2012. "JModelTest 2: More Models, New Heuristics and Parallel Computing." *Nature Methods* 9 (8): 772.

Davis, Nicole M., Diana M. Proctor, Susan P. Holmes, David A. Relman, and Benjamin J. Callahan. 2018. "Simple Statistical Identification and Removal of Contaminant Sequences in Marker-Gene and Metagenomics Data." *Microbiome* 6 (1): 226.

DeSantis, T. Z., P. Hugenholtz, N. Larsen, M. Rojas, E. L. Brodie, K. Keller, T. Huber, D. Dalevi, P. Hu, and G. L. Andersen. 2006. "Greengenes, a Chimera-Checked 16S RRNA Gene Database and Workbench Compatible with ARB." *Applied and Environmental Microbiology* 72 (7): 5069–72.

Duron, Olivier, Ulrich E. Schneppat, Arnaud Berthomieu, Steven M. Goodman, Boris Droz, Christophe Paupy, Judicaël Obame Nkoghe, Nil Rahola, and Pablo Tortosa. 2014. "Origin, Acquisition and Diversification of Heritable Bacterial Endosymbionts in Louse Flies and Bat Flies." *Molecular Ecology* 23 (8): 2105–17.

Eisenhofer, Raphael, Jeremiah J. Minich, Clarisse Marotz, Alan Cooper, Rob Knight, and Laura S. Weyrich. 2019. "Contamination in Low Microbial Biomass Microbiome Studies: Issues and Recommendations." *Trends in Microbiology* 27 (2): 105–17.

Fernandes, Andrew D., Jennifer Ns Reid, Jean M. Macklaim, Thomas A. McMurrough, David R. Edgell, and Gregory B. Gloor. 2014. "Unifying the Analysis of High-Throughput Sequencing Datasets: Characterizing RNA-Seq, 16S RRNA Gene Sequencing and Selective Growth Experiments by Compositional Data Analysis." *Microbiome* 2 (May): 15.

Folmer, O., M. Black, W. Hoeh, R. Lutz, and R. Vrijenhoek. 1994. "DNA Primers for Amplification of Mitochondrial Cytochrome c Oxidase Subunit I from Diverse Metazoan Invertebrates." *Molecular Marine Biology and Biotechnology* 3 (5): 294–99.

Gilbert, Jack A., Janet K. Jansson, and Rob Knight. 2014. "The Earth Microbiome Project: Successes and Aspirations." *BMC Biology* 12 (August): 69.

Gilbert, Jack A., Folker Meyer, Dion Antonopoulos, Pavan Balaji, C. Titus Brown, Christopher T. Brown, Narayan Desai, et al. 2010. "Meeting Report: The Terabase Metagenomics Workshop and the Vision of an Earth Microbiome Project." *Standards in Genomic Sciences* 3 (3): 243–48.

Glöckner, Frank Oliver, Pelin Yilmaz, Christian Quast, Jan Gerken, Alan Beccati, Andreea Ciuprina, Gerrit Bruns, et al. 2017. "25 Years of Serving the Community with Ribosomal RNA Gene Reference Databases and Tools." *Journal of Biotechnology* 261 (November): 169–76.

Gloor, Gregory B., Jean M. Macklaim, Vera Pawlowsky-Glahn, and Juan J. Egozcue. 2017. "Microbiome Datasets Are Compositional: And This Is Not Optional." *Frontiers in Microbiology* 8 (November): 2224.

Gloor, Gregory B., and Gregor Reid. 2016. “Compositional Analysis: A Valid Approach to Analyze Microbiome High-Throughput Sequencing Data.” *Canadian Journal of Microbiology* 62 (8): 692–703.

Hebert, Paul D. N., Alina Cywinska, Shelley L. Ball, and Jeremy R. deWaard. 2003. “Biological Identifications through DNA Barcodes.” *Proceedings of the Royal Society of London. Series B: Biological Sciences* 270 (1512): 313–21.

Hosokawa, Takahiro, Naruo Nikoh, Ryuichi Koga, Masahiko Satô, Masahiko Tanahashi, Xian-Ying Meng, and Takema Fukatsu. 2012. “Reductive Genome Evolution, Host–Symbiont Co-Speciation and Uterine Transmission of Endosymbiotic Bacteria in Bat Flies.” *The ISME Journal* 6 (3): 577–87.

Katoh, Kazutaka, Kei-Ichi Kuma, Hiroyuki Toh, and Takashi Miyata. 2005. “MAFFT Version 5: Improvement in Accuracy of Multiple Sequence Alignment.” *Nucleic Acids Research* 33 (2): 511–18.

Katoh, Kazutaka, Kazuharu Misawa, Kei-Ichi Kuma, and Takashi Miyata. 2002. “MAFFT: A Novel Method for Rapid Multiple Sequence Alignment Based on Fast Fourier Transform.” *Nucleic Acids Research* 30 (14): 3059–66.

Katoh, Kazutaka, and Hiroyuki Toh. 2007. “PartTree: An Algorithm to Build an Approximate Tree from a Large Number of Unaligned Sequences.” *Bioinformatics* 23 (3): 372–74.

Kearse, Matthew, Richard Moir, Amy Wilson, Steven Stones-Havas, Matthew Cheung, Shane Sturrock, Simon Buxton, et al. 2012. “Geneious Basic: An Integrated and Extendable Desktop Software Platform for the Organization and Analysis of Sequence Data.” *Bioinformatics* 28 (12): 1647–49.

Larkin, M. A., G. Blackshields, N. P. Brown, R. Chenna, P. A. McGettigan, H. McWilliam, F. Valentin, et al. 2007. “Clustal W and Clustal X Version 2.0.” *Bioinformatics* 23 (21): 2947–48.

McMurdie, Paul J., and Susan Holmes. 2012. “Phyloseq: A Bioconductor Package for Handling and Analysis of High-Throughput Phylogenetic Sequence Data.” *Pacific Symposium on Biocomputing. Pacific Symposium on Biocomputing*, 235–46.

———. 2014. “Waste Not, Want Not: Why Rarefying Microbiome Data Is Inadmissible.” *PLoS Computational Biology* 10 (4): e1003531.

Miller, M. A., W. Pfeiffer, and T. Schwartz. 2010. “Creating the CIPRES Science Gateway for Inference of Large Phylogenetic Trees.” In *2010 Gateway Computing Environments Workshop (GCE)*, 1–8. ieeexplore.ieee.org.

Nováková, Eva, Václav Hypsa, and Nancy A. Moran. 2009. “Arsenophonus, an Emerging Clade of Intracellular Symbionts with a Broad Host Distribution.” *BMC Microbiology* 9 (July): 143.

Parada, Alma E., David M. Needham, and Jed A. Fuhrman. 2016. “Every Base Matters: Assessing Small Subunit RRNA Primers for Marine Microbiomes with Mock Communities, Time Series and Global Field Samples.” *Environmental Microbiology* 18 (5): 1403–14.

Price, Morgan N., Paramvir S. Dehal, and Adam P. Arkin. 2010. “FastTree 2--Approximately Maximum-Likelihood Trees for Large Alignments.” *PloS One* 5 (3): e9490.

Quast, Christian, Elmar Pruesse, Pelin Yilmaz, Jan Gerken, Timmy Schweer, Pablo Yarza, Jörg Peplies, and Frank Oliver Glöckner. 2013. “The SILVA Ribosomal RNA Gene Database Project: Improved Data Processing and Web-Based Tools.” *Nucleic Acids Research* 41 (Database issue): D590-6.

Rambaut, A. 2014. "FigTree 1.4. 2 Software." *Institute of Evolutionary Biology, Univ. Edinburgh*.

Rambaut, A., and A. J. Drummond. 2012. "FigTree Software."

Silverman, Justin D., Alex D. Washburne, Sayan Mukherjee, and Lawrence A. David. 2017. "A Phylogenetic Transform Enhances Analysis of Compositional Microbiota Data." *ELife* 6 (February). https://doi.org/10.7554/eLife.21887.

Stamatakis, Alexandros. 2014. "RAxML Version 8: A Tool for Phylogenetic Analysis and Post-Analysis of Large Phylogenies." *Bioinformatics* 30 (9): 1312–13.

Thompson, J. D., D. G. Higgins, and T. J. Gibson. 1994. "CLUSTAL W: Improving the Sensitivity of Progressive Multiple Sequence Alignment through Sequence Weighting, Position-Specific Gap Penalties and Weight Matrix Choice." *Nucleic Acids Research* 22 (22): 4673–80.

Trowbridge, Richard E., Katharina Dittmar, and Michael F. Whiting. 2006. "Identification and Phylogenetic Analysis of Arsenophonus- and Photorhabdus-Type Bacteria from Adult Hippoboscidae and Streblidae (Hippoboscoidea)." *Journal of Invertebrate Pathology* 91 (1): 64–68.

Tsilimigras, Matthew C. B., and Anthony A. Fodor. 2016. "Compositional Data Analysis of the Microbiome: Fundamentals, Tools, and Challenges." *Annals of Epidemiology* 26 (5): 330–35.

Xia, Yinglin, and Jun Sun. 2017. "Hypothesis Testing and Statistical Analysis of Microbiome." *Genes & Diseases* 4 (3): 138–48.

Yilmaz, Pelin, Laura Wegener Parfrey, Pablo Yarza, Jan Gerken, Elmar Pruesse, Christian Quast, Timmy Schweer, Jörg Peplies, Wolfgang Ludwig, and Frank Oliver Glöckner. 2014. "The SILVA and 'All-Species Living Tree Project (LTP)' Taxonomic Frameworks." *Nucleic Acids Research* 42 (Database issue): D643-8.

Table S1: Patch area, isolation, and distance from source (REGUA).

Fragment ID	Area	Isolation	Distance From Source
F1	21.15	600	3748.024
F2	34.11	234.31	5453.124
F3	41.04	84.85	3142.76
F4	52.11	362.49	6557.559
F5	84.33	150	8520.907
F6	92.34	210	2569.115
F7	99.99	349.86	6753.698
F8	117.27	134.16	357.26
F9	184.77	174.93	7525.56
F10	228.78	480	405.114
REGUA	62378.64	60	NA
REGUA2	62378.64	60	NA
REGUA3	62378.64	60	NA

Table S2: PERMANOVA on well-sampled species showing the p-value (top; *=p<0.05, **=p<0.005, ***=p<0.0005), R2 (middle) and p-value for homoscedasticity (bottom, significance indicates violation of the assumptions of PERMANOVA).

	Paratrichobius longicrus	***Speiseria ambigua***	***Strebla guajiro***	***Trichobius joblingi***
	0.8050	0.0820	0.8723	0.0091**
**Parasite Sex**	0.0164	0.2128	0.0254	0.2513
	0.5820	0.0070**	0.9680	0.0110*
	0.7014	0.8746	0.0573	0.5582
**Bat Sex**	0.0249	0.0128	0.3233	0.0206
	0.9070	0.0790	0.0001***	0.5070
	0.6494		0.5742	0.6700
**Protection Status**	0.0133		0.0314	0.0073
	0.8320		0.5490	0.8750
	0.6373	0.4681	0.6516	0.5989
**Log$_2$ Area**	0.0139	0.0391	0.0248	0.0145
	0.5110	0.0630	0.4260	0.6850
	0.7385	0.7440	0.1665	0.6143
**Log$_2$ Isolation**	0.0106	0.0124	0.1042	0.0120
	0.4870	0.0580	0.4180	0.7210
	0.2919	0.9885	0.8427	0.1096
**Sampling Site**	0.2836	0.2705	0.2924	0.5395
	0.4750	0.0710	0.4930	0.6240

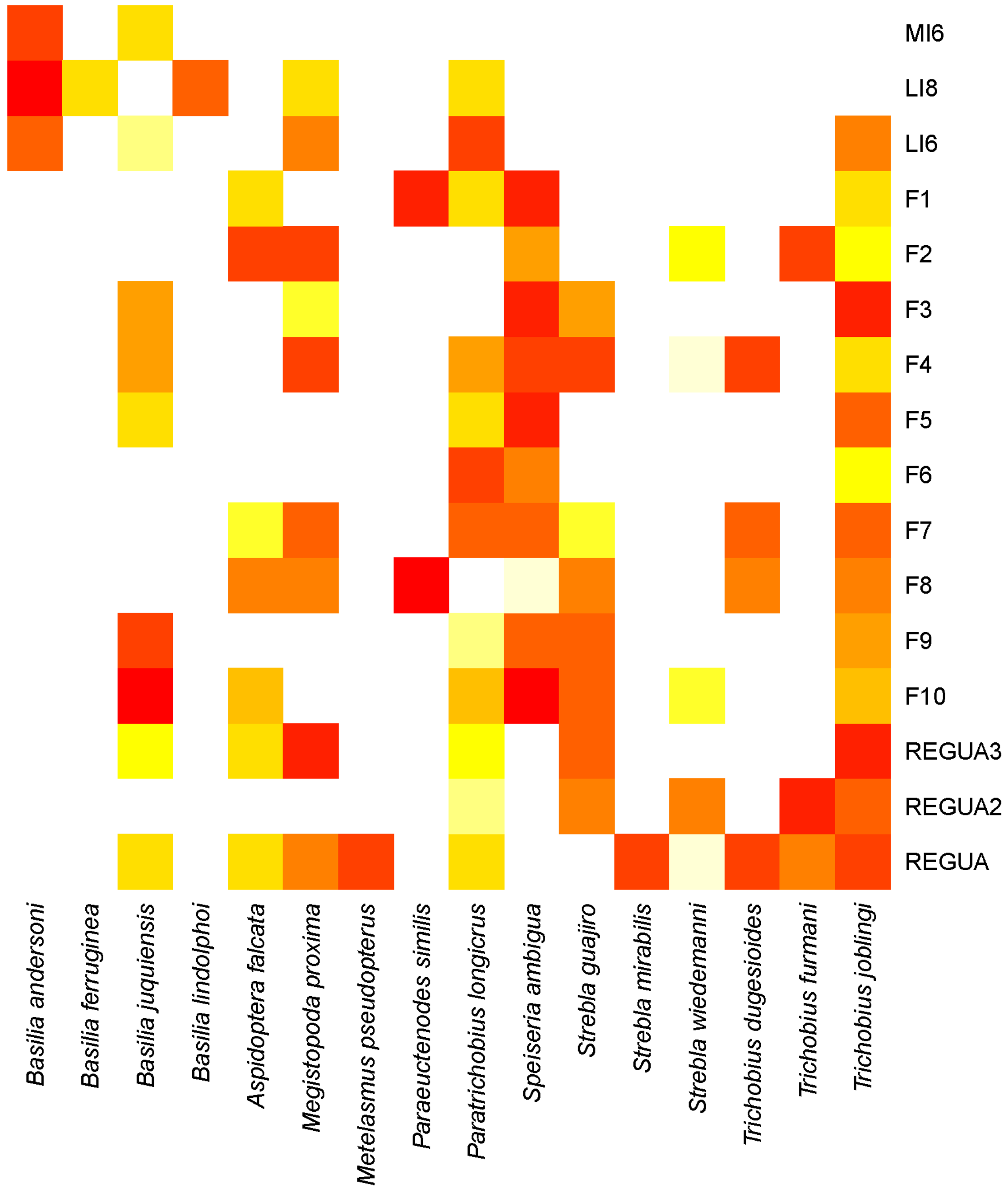

Figure S1: Heatmap shows distribution of parasite species across the sampled sites, where white means no samples were collected, red means ~1 parasite individual was collected, and pale yellow means ~5 parasite individuals were collected.

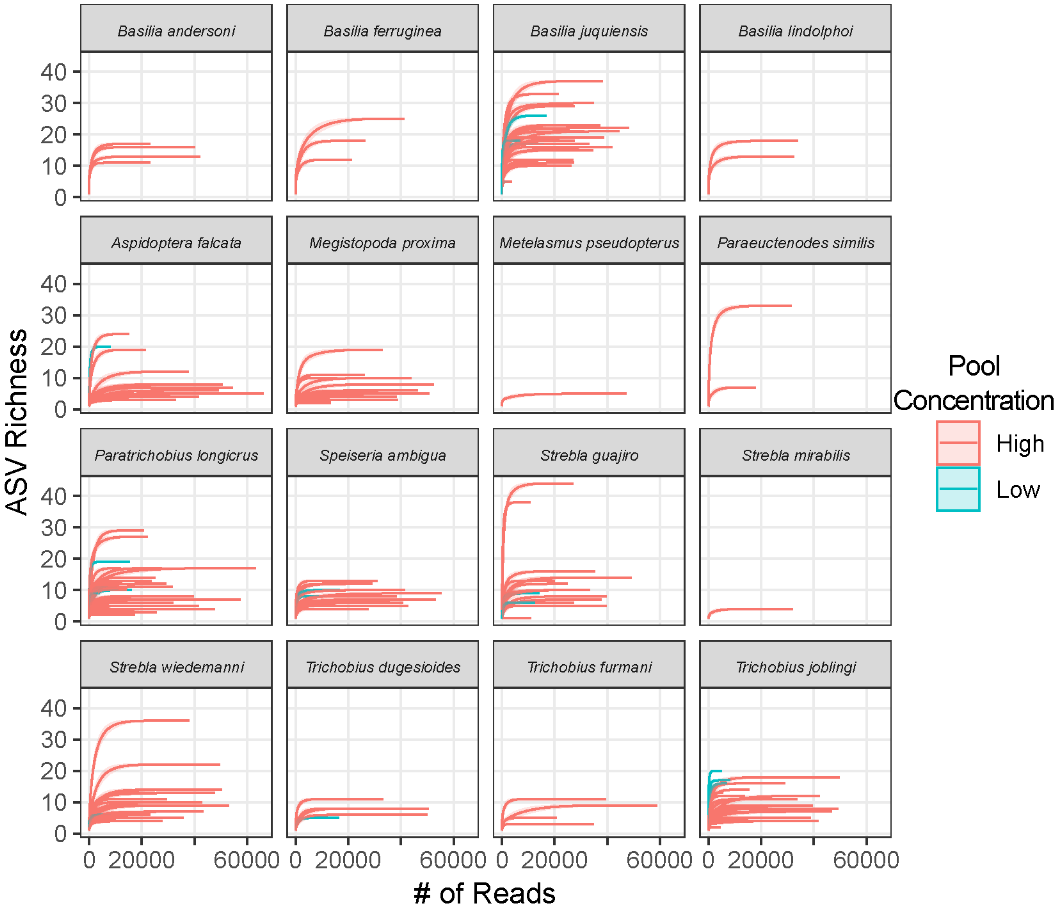

Figure S2: Rarefaction curves of bacterial ASVs detected at various sequencing depths in each parasite species. Each line represents a sample. Red lines are high concentration samples and blue lines are low concentration samples. Reads were removed from a sample if they were present at less than 0.01% relative abundance.

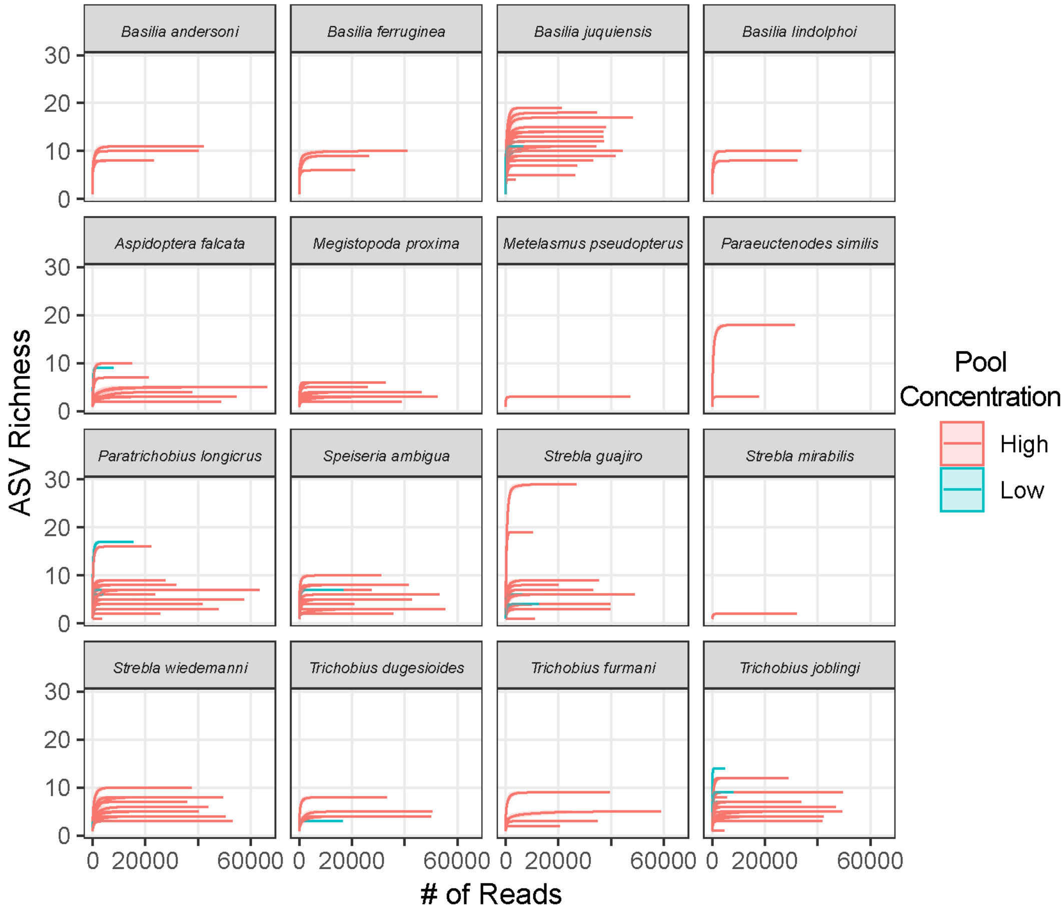

Figure S3: Rarefaction curves of bacterial ASVs detected at various sequencing depths in each parasite species. Each line represents a sample. Red lines are high concentration samples and blue lines are low concentration samples. Reads were removed from a sample if they were present at less than 0.1% relative abundance.

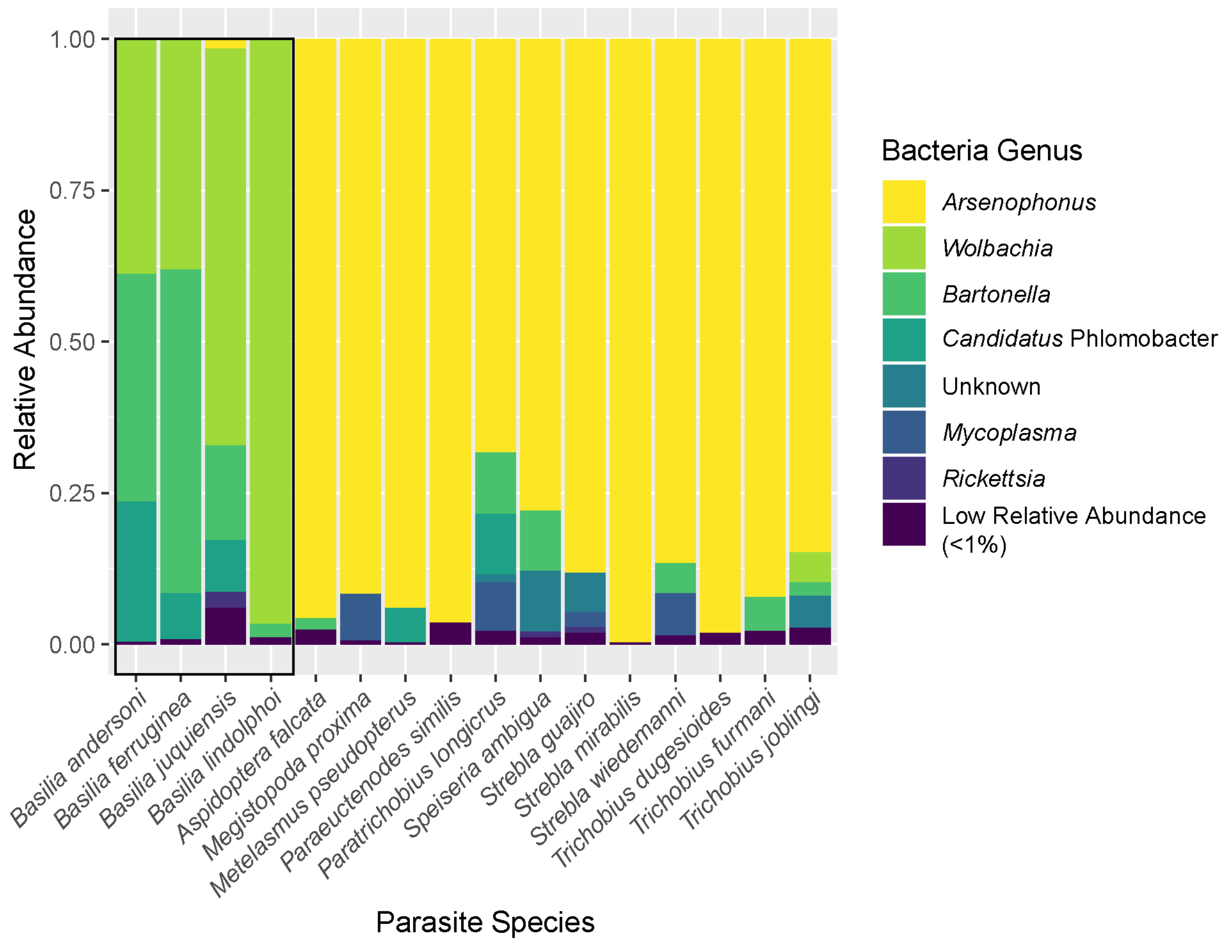

Figure S4: Relative abundance of bacterial genera in each parasite species, when reads were removed from a sample if they were present at less than 0.1% relative abundance. Nycteribiidae are within the black box and Streblidae are outside of the black box.

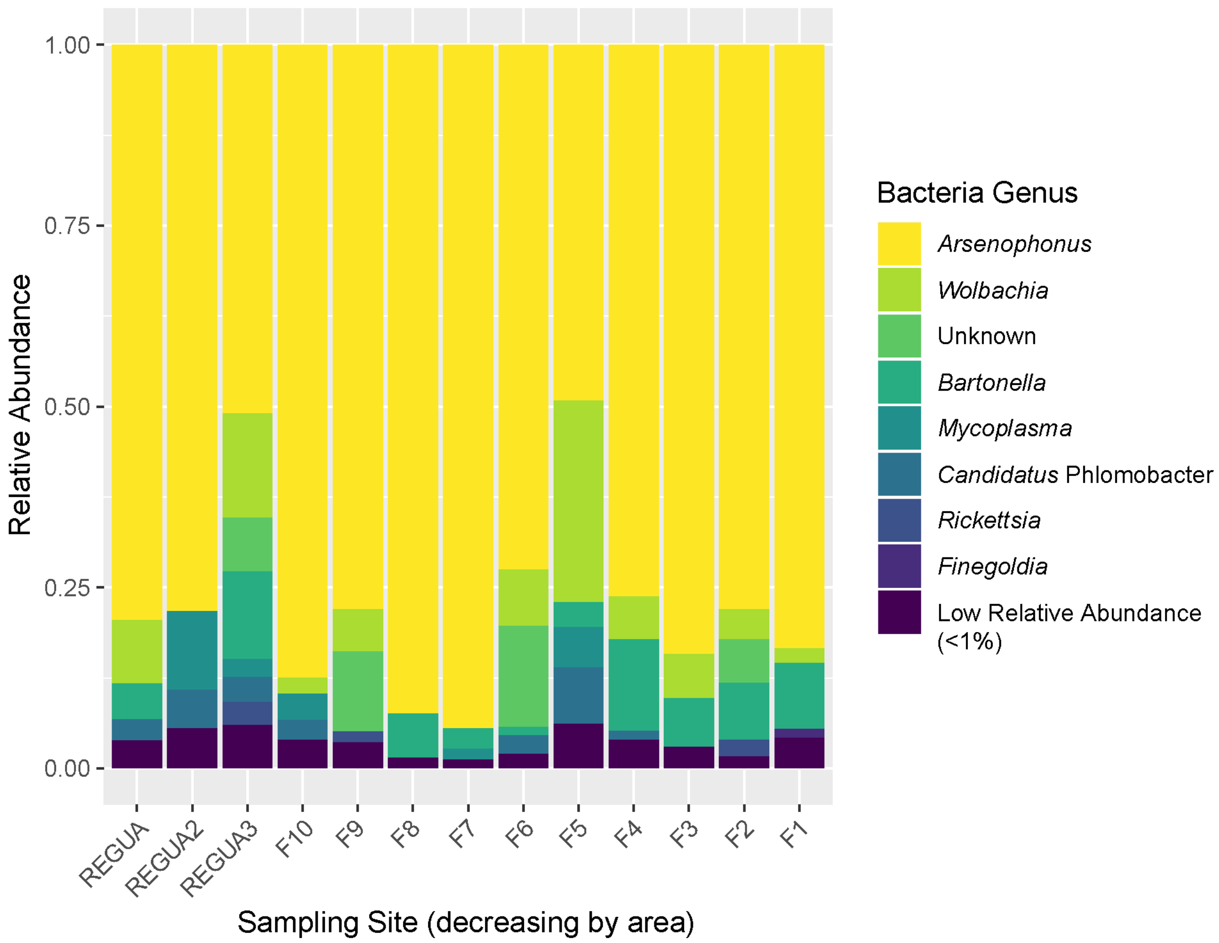

Figures S5: Relative abundance of bacterial genera at each sampling site.

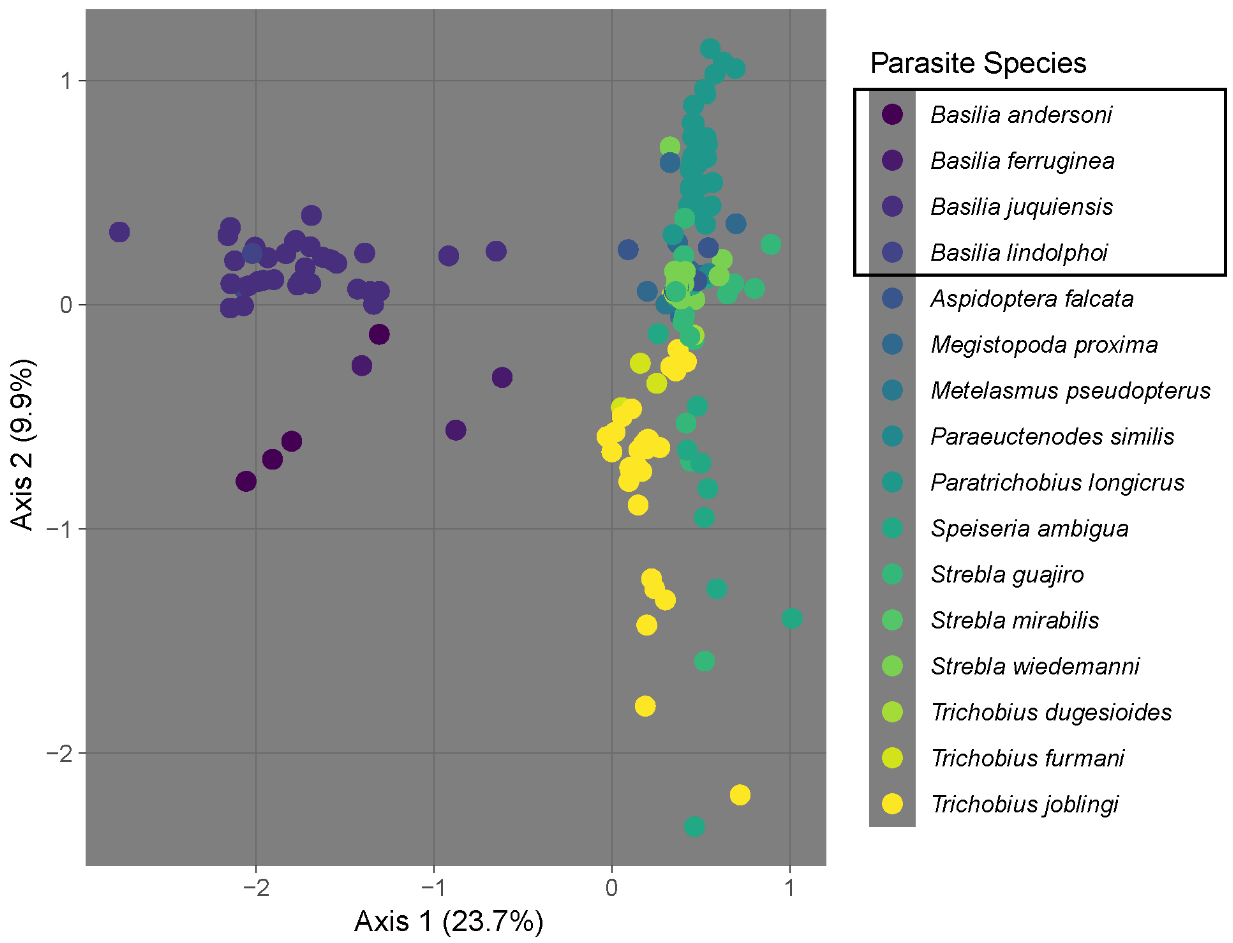

Figures S6: Principal coordinates analysis of samples when were removed from a sample if they were present at less than 0.1% relative abundance. Colors indicate parasite species.

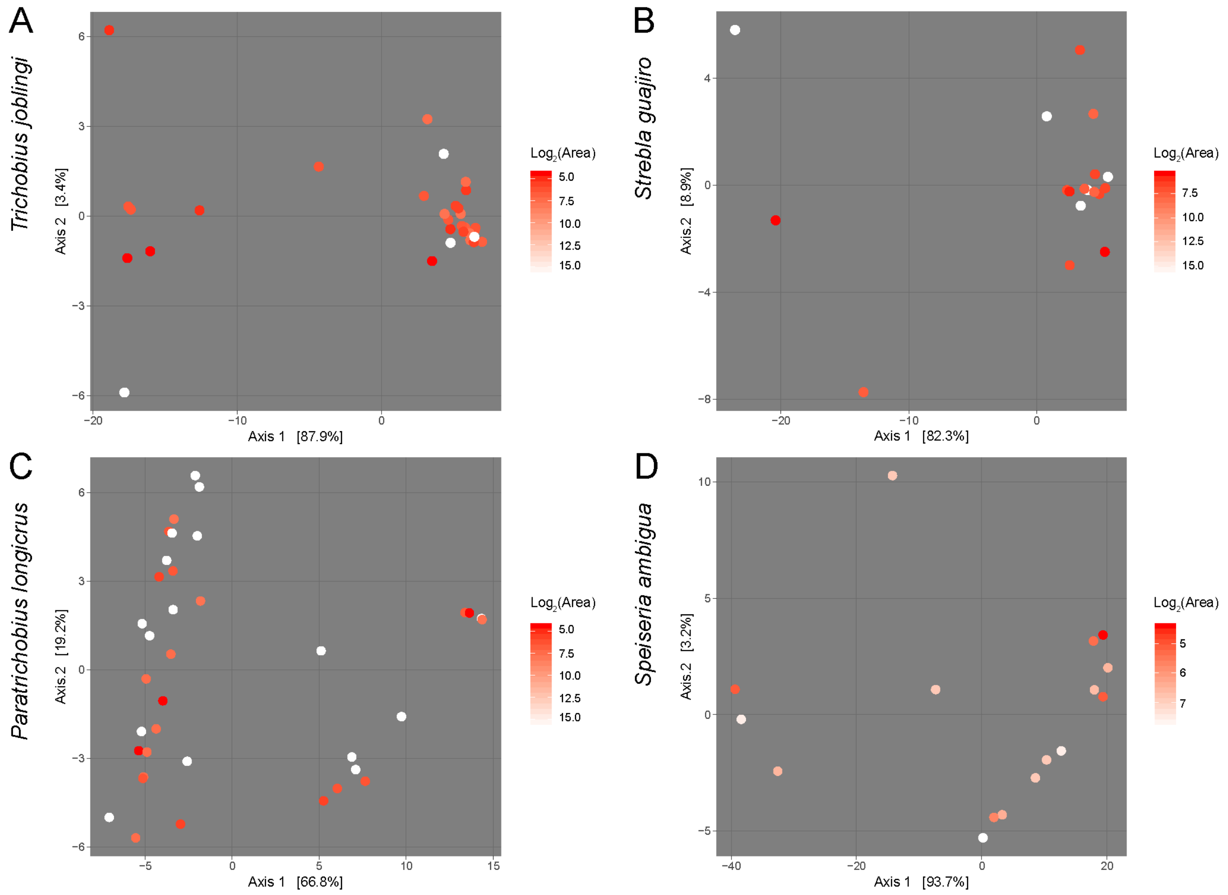

Figure S7: Principal Coordinates Analysis on the Euclidean distances between philr-transformed microbial abundances of each of the four most well-sampled bat fly species (A-D). Each species is plotted with distance to source colored from white (near) to red (far) and separately with habitat fragment area colored white (large) to red (small).

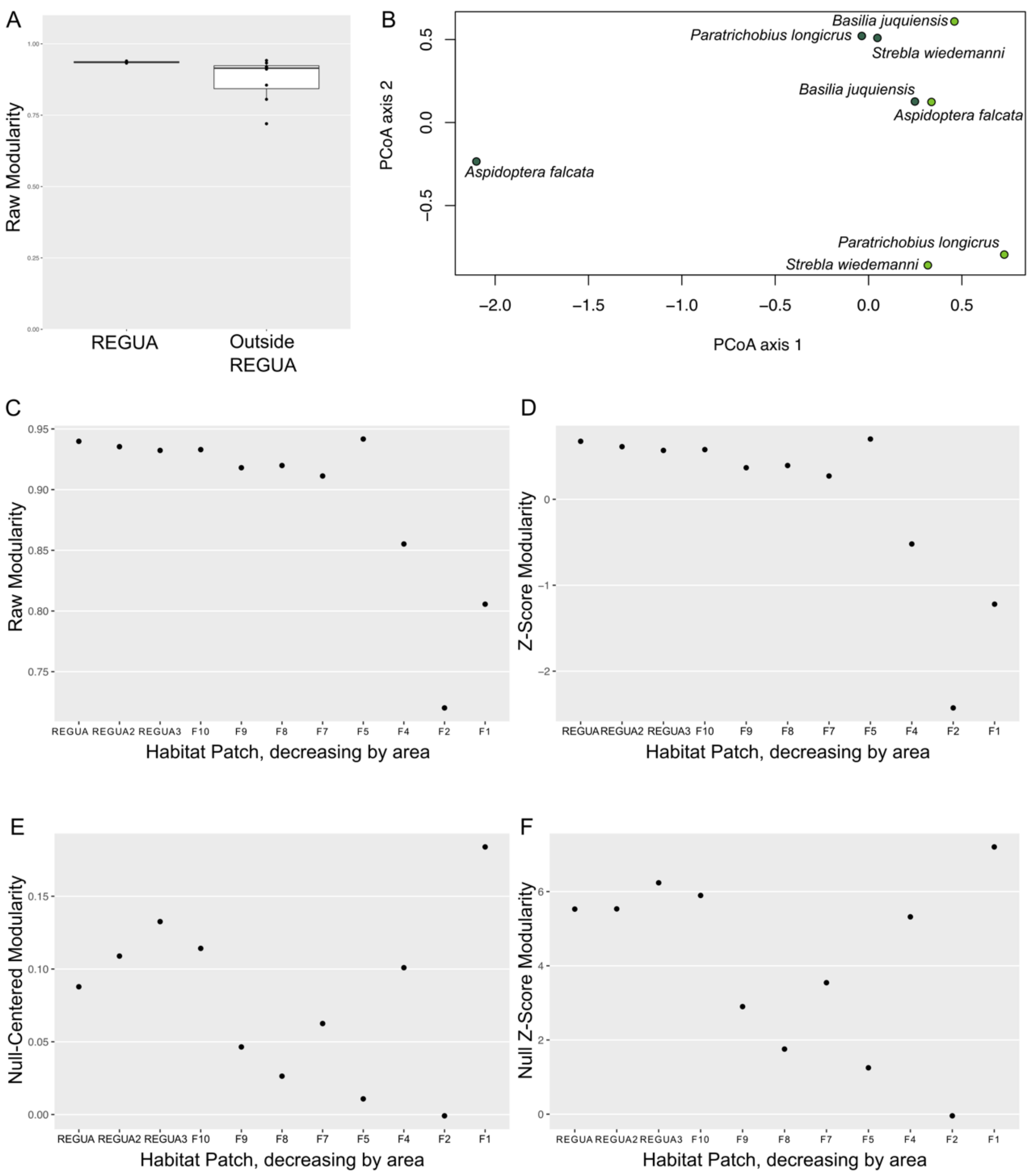

Figure S8: (A) Raw modularity between networks from within and outside of REGUA. (B) PCoA of orbit distributions of within-species networks. Lime green dots indicate networks outside of REGUA and dark green indicated within-REGUA networks. Parasite species names are provided next to each point. (C) Raw modularity of habitat patch networks decreasing by area. (D) Z-score modularity of habitat patch networks decreasing by area. (E) Null-centered modularity of habitat patch networks decreasing by area. (F) Modularity of habitat patch networks centered and scaled by the their null distributions.
